# Chemistry of the Metals—Mercury

**Published:** 1855-01

**Authors:** Reginald N. Wright


### THE AMERICAN JOURNAL OF DENTAL SCIENCE.

Vol. V. NEW SERIES—JANUARY, 1855. No. 1.

### ORIGINAL COMMUNICATIONS.

### ARTICLE I.

*Chemistry of the Metals—Mercury.* By Professor REGINALD N. WRIGHT, A. M., M. D.

(Continued from page 515.)

*Proto-sulphuret of Mercury.*—To prepare a true and pure proto-sulphuret of mercury, an excellent method is to pass a current of sulphureted hydrogen gas, through a solution of the *proto-nitrate;* precipitation ensues freely, and the result after subsidence must be thrown on a filter and carefully dried: again, triturate well three parts of sulphur with one of mercury, for a sufficient time and the result will be a black mass, which is almost destitute of taste, but which on being boiled with caustic potash, seems to be a mixture of the bisulphuret and sulphur. Taddei gives the following process for the production of "Ethop's mineral:" triturate in a mortar until the whole becomes a dark colored paste, a small quantity of water, three parts of mercury and one of sulphuret of potassium, after adding "sulphur sublimatum," in weight equal to the mercury used, dissolve out the alkaline sulphuret with repeated washings and dry the residue.

When prepared according to the first method, Brand says, "if it is boiled with nitric acid, it is converted into a sulphate of mercury; heated to redness, metallic mercury escapes, and bisulphuret sublimes. By long exposure to light, it is said to be resolved into mercury and bisulphuret. It consists, according to Guibourt (Ann. de ch. et Ph. i,) of 100 mercury+8.2 sulphur, number, which correspond to"

| | | | |
|---|---|---|---|
| Mercury, . . . | 1 | 200 | 92.6. |
| Sulphur, . . . | 1 | 16 | 7.4. |
| Proto-sulphuret of mercury, | 1 | 216 | 100.0. |

*Bisulphuret of Mercury or Cinnabar*—Is a compound having a beautiful red color, and is extensively used as a pigment; though sometimes found native, it is most beautiful when carefully prepared artificially, and in its preparation, the *Chinese* have been eminently successful. To prepare it, mix intimately sulphur and mercury, in the proportions of one part of the former to six of the latter, in an iron vessel, apply heat, and agitate constantly; the result must be heated to redness over a sand bath; sublimation ensues, and when cold the mass must be carefully rubbed in a mortar.

If a stream of sulphureted hydrogen gas is passed through a solution of the corrosive chloride of mercury in water, the bisulphuret is produced, but it must be sublimed and pulverized finely before it acquires the proper color, for when first formed, it is very dark, presenting nothing externally of the appearance of cinnabar.

It is not well to heat together *large* quantities of mercury and sulphur, for at the moment of combination, there is produced a considerable amount of action, almost explosive, much light and heat attending the union, accompanied with the elimination of sulphureted hydrogen gas.

By whatever process formed, it remains unchanged by simple exposure to the air for any length of time; but, if chemical action or heat be brought to bear upon it, decomposition speedily results. When heat (as in distillation) associated with the alkalies or alkaline earths acts upon it, decomposition ensues, and if heated even to dull redness, exposed to the action of the air, the same result follows, sulphurous acid and mercury escaping in vapor.

The following, from high authority, is not without interest in this place:* "Kincluff was the first who pointed out a mode of obtaining vermilion by the process of triturating mercury and sulphur in a solution of potassa. (Nicholson's Journal, 4to ii.) A black compound is first formed, and the mixture is then heated to 130°, and retained for several hours at about that temperature, when it gradually acquires a brown, and then a red tint; when this is sufficiently brilliant, the liquid is decanted, and the product washed and dried. Liebig, obtains vermilion by digesting recently prepared white precipitate in a sulphuret of ammonium, obtained by saturating hydrosulphuret of ammonia with sulphur; the black sulphuret is first produced, which afterwards acquires the desired red color, its tint is improved by digestion at a gentle heat in a strong solution of potassa." Bisulphuret of mercury consists of mercury 1—sulphur 2—its number being 232.

When carefully prepared, it can be volatilized without residuum, but when mixed with red metallic oxyds, as red oxyd of lead, it having a residuum on the application of heat.

† "Chlorosulphuret of mercury, (hg+2c)+2(hg+2s.) When sulphureted hydrogen gas is passed through a solution of corrosive sublimate, a white precipitate is first formed, which is long in subsiding, and readily passes through filtering paper; if the action of the gas be continued, this white compound blackens and becomes bisulphuret of mercury. The same white compound is formed by precipitating bisulphuret of mercury, in a solution of corrosive sublimate. It may be washed and dried without decomposition; when heated, corrosive sublimate rises first, and afterwards bisulphuret of mercury; it is insoluble in the greater number of acids, but nitro-hydrochloric acid decomposes it, chlorine converts it into chloride of sulphur and perchloride of mercury; the alkalies blacken it, and forming chlo-

* Brande, p. 944. † Brande, p. 945.

rides of their bases, separate sulphur and peroxyd of mercury. It consists of"

| | | | |
|---|---|---|---|
| Bisulphuret of mercury, | 2 | 464 | 63.1. |
| Perchloride of mercury, | 1 | 272 | 36.9. |
| | — | — | — |
| | 1 | 736 | 100.0. |

"*Iodosulphuret of Mercury*—(hg+2i) + 2 (hg+2s) is an analogous compound to the preceding, obtained by the action of sulphureted hydrogen, upon biniodide of mercury, it is of a yellow color."

"*Bromosulphuret*, (hg+2b) + 2 (hg+2s) is yellowish white and resolved by heat into bromide of mercury, which is most volatile, and into sulphuret, which subsequently sublimes."

"*Fluosulphuret of Mercury*—(hg+2f) + 2 (hg+2s)—When a solution of peroxyd of mercury, in hydrofluoric acid is subjected to the action of sulphureted hydrogen, a white precipitate is formed, which is characteristically distinguished from the preceding combinations, by its being decomposed by boiling water into sulphuret of mercury and perfluoride of mercury. When heated with S. acid, hydrofluoric acid is disengaged."

*Chloride of Mercury.*—This substance enters into combination with chlorine gas, in two proportions, producing calomel and corrosive sublimate, (as they are sometimes designated.) We shall not advocate the view which considers the black oxyd as a dioxyd and call the first a dichloride and the second a protochloride—but, call calomel protochloride, and corrosive sublimate bichloride of mercury.

According to Leopold Gmelin, 1. "Mercury slowly absorbs chlorine gas at ordinary temperatures and produces a gray pulverulent mass of mercury and dichloride; when introduced into chlorine gas at a boiling heat, it burns with a yellowish-red flame, producing dichloride and protochloride of mercury. 2. Mercury, electrified for a considerable time, in contact with hydrochloric acid gas, separates the hydrogen, and is itself converted into calomel. 3. Mercury agitated with aqueous sesqui-chloride of iron, yields calomel and aqueous protochloride of iron; the action is accelerated by the presence of free hydrochloric acid, (Schaffhäutl.) 4. Calomel is likewise formed on bringing mercurious oxyd in contact with hydrochloric acid, or a mercurious salt with the chloride of an alkali-metal. 5. On heating a mixture of mercury and the protochloride."

The following is transcribed verbatim from Mr. Brande, for two reasons—1st, because the author believes it to be the best account (extant) of the substances in question; and 2dly, because, the work from which the extract is made, is not within the reach of the majority of our readers.

He says, "There are two processes by which calomel is usually obtained; the one by the action of mercury on corrosive sublimate; the other, by the mutual decomposition of protosulphate of mercury and chloride of sodium; the product is in both cases, sublimed by heat. The first mode consists in triturating four parts of corrosive sublimate with three of mercury, (and a little water to prevent the dust rising,) until the globules disappear, and the whole assumes the appearance of an homogenious gray powder, which is introduced into a matrass, placed in a sand heat, and gradually raised to redness. The protochloride sublimes, mixed with a little perchloride, the greater part of which, however, being more volatile than the calomel, rises higher in the matrass; that which adheres to the protochloride, may be separated by reducing the whole to a fine powder, and washing in large quantities of distilled water, hot. Pure protochloride of mercury, in the form of a yellowish-white, insipid powder remains. It was formerly the custom to submit this product, to very numerous sublimations, under the idea of rendering it mild; but these often tended to the production of corrosive sublimate, and the calomel of the first sublimation, especially if a little excess of mercury be found in it, is often more pure than that furnished by subsequent operations.

"In this process, the operation consists in reducing the perchloride, to the state of protochloride, by the addition of mercury; it is objectionable on account of the heat required, and the destruction of the matrasses in which it is sublimed, so that some have proposed to substitute the humid process, as it is termed, by precipitation, as proposed by Scheele and Chevenix.

It is as follows: Form a protonitrate of mercury by dissolving as much mercury as possible in nitric acid, then dissolve in boiling water, a quantity of common salt, equal to half the weight of the mercury used, and render this solution sensibly sour by hydrochloric acid, and pour the hot nitrate of mercury into it. Wash and dry the precipitate. If this process be carefully performed, and the precipitate be thoroughly edulcorated, the calomel is said to be sufficiently pure; but a small portion of chloride of sodium remains, combined with the calomel, which material affects its medical uses.

"The second process, by sublimation, however, appears on the whole, the least objectionable for the production of this important article of pharmacy. It is as follows: boil 2 pounds of mercury with 30 ounces of sulphuric acid, in a glass vessel until the sulphate of mercury is dry. When it has cooled, rub it with 2 pounds of mercury in an earthenware mortar, until they are well mixed, then add 1½ pounds of chloride of sodium, and rub them together until globules are no longer visible. Put this mixture into a proper vessel, and heat it gradually to redness, the protochloride of mercury, or calomel, sublimes and condenses in various forms, according to the rapidity of the operation, and the form and capacity of the subliming vessel. If intended for medicinal use, it should be cautiously reduced to an impalpable powder, and washed with repeated affusions of hot distilled water until it becomes perfectly tasteless, and the water in which it has been washed is not discolored by the addition of an aqueous solution of sulphureted hydrogen.

"This process has many advantages over the first method. The sulphate of mercury may be formed by boiling the metal with sulphuric acid, to dryness, in a cast iron vessel, which should be conveniently arranged, for the escape of the abundant fumes of sulphurous acid developed by the action of the mercury, and which are often a serious nuisance to the neighborhood. They may be very effectually annihilated, by suffering them to pass through a long flue and lofty chimney, mixed with abundance of coal smoke. The persulphate of mercury is triturated with a sufficient quantity of metallic mercury, to convert it into a protosulphate, and then mixed with a due proportion of common salt, and subjected to sublimation.

"Protosulphate of mercury is a compound of,

| | | |
|---|---|---|
| 1. | Proportional of protoxyd of mercury, . | 208 |
| 1. | " " sulphuric acid, . . . . | 40 |
| | Total, . . . . . | 248 |

To convert it into protochloride of mercury, it is mixed with 1 proportional of chloride of sodium, the chlorine of which combines with the mercury of the oxyd of mercury in the protosulphate, to form a protochloride, while its sodium becomes sulphate of soda, as shown in the following diagram, in which, the original compounds are printed in small capitals, the products in italics, and the component substances, in common type; the equivalent numbers are affixed to the respective agents.

"In washing calomel, sal ammoniac is often used, in consequence of the extreme solubility which it confers on the perchloride of mercury: common salt answers equally well, and is cheaper; but it deserves notice in relation to this part of the process, that calomel boiled with solution of sal ammoniac, or of common salt, is resolved into metallic mercury and corrosive sublimate; the washings, therefore, where these salts are employed, should be with their cold solutions, but it is safer to wash with hot distilled water only, which, when conjoined with perfect levigation, and tested as above directed, is very effective.

*Protochloride of mercury,*
236.

| | | |
|---|---|---|
| | Mercury + Chlorine,<br>200. 36. | |
| 248.<br>PROTO-<br>SULPHATE<br>OF<br>MERCURY. | Sulphuric<br>Acid 40.<br>+<br>Oxygen 8·+Sodium 24. | 60.<br>CHLORIDE OF<br>SODIUM. |

*Sulphate of Soda,*
72.

"The form in which calomel sublimes, depends much upon the dimensions and temperature of the subliming vessels. In small vessels it generally condenses in a crystalline cake, the interior surface of which is often covered with beautiful quadrangular crystals, prismatic in form, (Brooke Ann. of Phil. ii, 427, 2d series,) transparent, and of a texture somewhat elastic or horny; in this state, it acquires, by the necessary rubbing into powder, a decidedly yellow or buff tint, more or less deep, according to the degree of trituration which it has undergone. If, on the contrary, the calomel be sublimed into a very capacious and cold receiver, it falls in a most impalpable and perfectly white powder, which only requires due elutriation to fit it for use, it then remains perfectly colorless. By a modification of the process it may be suffered as it sublimes to fall into water, according to Mr. Jewell's patent.

"The above circumstances, too, account for the various appearances under which calomel occasionally presents itself in commerce; it may be added, that the buff aspect of this substance indicates the absence of corrosive sublimate, though it by no means follows, as a consequence, that when snow-white, it contains it. When the surface of massive sublimed calomel is scratched, it always exhibits a buff color; it also becomes yellow when heated, but loses this tint as it again cools.

Calomel should be perfectly tasteless, inodorous and insoluble in water. Dumas states it to be soluble in 12,000 parts of boiling water, and Graham says it is so insoluble, that when protonitrate of mercury is added to water containing a $\frac{250}{1000}$th part of hydrochloric acid, a sensible precipitate of calomel appears. I have found that upon pouring hot distilled water on pure calomel on a good filter, the liquor which passes, is not affected by sulphureted hydrogen. Its specific gravity is 7-17 (Hassenfratz,) 6-5, (Graham.) At a heat somewhat below redness, it rises in vapors, without previous fusion; but it fuses when subjected to heat under pressure. The density of its vapors is 8-2, or 119, in reference to hydrogen as unity. When scratched, or broken in the dark, it phosphoresces. It is decomposed by the fixed alkalies and by ammonia, and protoxyd of mercury, is one of the results. Ammoniacal gas blackens calomel, but heat restores the original color, and the calomel is unchanged. By strong hydrochloric acid, it is resolved into mercury and corrosive sublimate, and by nitric acid, into pernitrate of mercury and corrosive sublimate. Phosporous, sulphur and several of the metals, decompose it. It consists of,

| | | | | |
|---|---|---|---|---|
| Mercury, . . | 1 | 200 | 84.75 | 85 |
| Chlorine, . . | 1 | 36 | 15.25 | 15 |
| Protochloride of mercury, | 1 | 236 | 100.00 | 100* |

*Native Protochloride of Mercury*, or *Mercurial Horn Ore*—has been found in Germany, France and Spain, usually crystallized, and sometimes incrusting and massive; it is rare.

*Perchloride of Mercury. Bichloride of Mercury, Oxymuriate of Mercury. Corrosive Sublimate.* (Hg+2c.)—When mercury is heated in excess of chlorine it burns with a pale flame, the gas is absorbed, and a white volatile substance arises, which is the *perchloride.* It may also be obtained by dissolving peroxyd of mercury in hydrochloric acid, evaporating to dryness, redissolving in water, and crystallizing.

The ordinary process for making corrosive sublimate, consists in exposing a mixture of chloride of sodium and persulphate of mercury to heat, in a proper subliming vessel. The persulphate is formed by boiling two pounds of mercury with thirty ounces of sulphuric acid to dryness; it is then rubbed to powder, with four pounds of chloride of sodium, and the mixture put into a large flask, or into an earthen subliming vessel, and exposed to a heat gradually raised to redness.

In this process, the original substances are decomposed, *perchloride of mercury* sublimes, and sulphate of soda is the residue; the object then, of the operation is, to obtain a compound of one proportional of mercury and two of chlorine, which, is effected by the mutual decomposition of one proportional of persulphate of mercury, $=296$, and two proportionals of chloride of sodium, $60\times2=120$, as shown in the annexed diagram:

*Turner. Davy. Zaboada.

Perchloride of mercury,
272.

| | | |
|---|---|---|
| 296. Per-sulphate of mercury. | Mercury + Chlorine.<br>200. 72.<br><br>Sulphuric Acid,<br>80.<br>+<br>Oxygen 16+Sodium 48 | 120. Chloride of sodium. |

Sulphate of Soda,
144.

The *Persulphate of Mercury*, is generally prepared upon the large scale, by heating the acid and metal in an iron pot, proper means being adopted to carry off the copious fumes of sulphurous acid, arising from the decomposition of a portion of the sulphuric acid, during the peroxydizement of the mercury. The whole is then evaporated to dryness, and the subsequent sublimation is performed in glass, earthenware or iron vessels, their form and arrangement being much dependent upon the quantity of materials employed.

Perchloride of mercury has an acrid, nauseous taste, leaving a permanent metallic and astringent flavor upon the tongue. Its specific gravity is 5·2 (Hassenfratz,) 6·5 (Graham.) It is usually met with in the shops in the form of white, semi-transparent and imperfectly crystallized masses, or in powder. It frequently exhibits prismatic crystals upon the inner surfaces of the sublimed cakes, (Brooke, Ann. of Phil., 2d series, vi, 285.) It is soluble in about 16 parts of cold and 3 of hot water, and as the solution cools, it deposits quadrangular crystals, which are prismatic; it is more copiously soluble in alcohol, 7 parts of which at 60° dissolve 3, and at its boiling point 6 of corrosive sublimate; it is also soluble in ether, which, at common temperatures, takes up about one-third its weight, and when ether is agitated with the aqueous solution, it abstracts the perchloride from the water. When heated, it readily fuses, boils and entirely sublimes in the form of a dense white vapor, powerfully affecting the nose and mouth. The density of its vapor is 9·420; 1 volume of it, including 1 volume of mercury vapor and 1 volume of chlorine. It is insoluble in concentrated nitric and sulphuric acids. Hydrochloride acid of the specific gravity of 1·158, at the temperature of 60° dissolves about its own weight, and the solution, when cooled to about 40°, concretes into a mass of acicular crystals; there appear to be two or three of these *definite hydrochlorates of bichloride of mercury;* they are partially decomposed when added to great excess of water, and resolved into free hydrochloric acid, and bichloride.

When solution of *perchloride of mercury* is decomposed by potassa, soda or lime, a yellow precipitate is thrown down, which, when the alkali is in excess, is a *hydrated-peroxyd of mercury.* Such a mixture of a pint of lime water, with a drachm of corrosive sublimate, was formerly much used as an application to venereal ulcers, under the name of *aqua-phagedœnica,* or *red lotion.* It is, in fact, a solution containing undecomposed corrosive sublimate and chloride of calcium, mixed with peroxyd, or with oxychloride of mercury. For the actual decomposition of 1 proportional of corrosive sublimate, 2 proportionals of lime are required, as shown in the annexed diagram, which also exhibits the theory of the decomposition.

2. Proportionals of chloride of calcium.
$56 \times 2 = 112.$

Chlorine 72 + Calcium 40.

1. Proportional of perchloride of mercury=272. | water. | 2. Proportionals of lime = $28 \times 2 = 56.$

Mercury + Oxygen.

200. 16.

1. Proportional of peroxyd of mercury.
=216.

When *protochloride of mercury,* or *calomel,* is similarly decomposed, a portion of *protoxyd of mercury is the result,* as in the annexed diagram, and the mixture, which is known in pharmacy under the name of *black lotion*, is obtained.

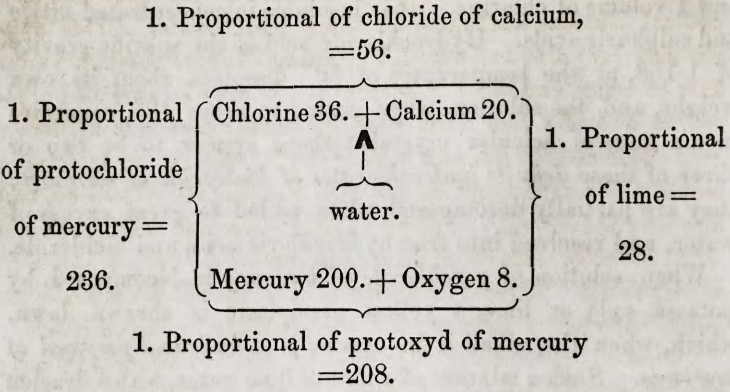

Corrosive sublimate is either decomposed by, or combines with, the greater number of organic bodies; some of them, convert it into calomel, by the abstraction of one atom of chlorine, others enter into more complicated combinations, some of which are remarkably permanent; the applications of it to the preservation of some anatomical preparations, and to the prevention of *dry-rot*, illustrate these actions; hence, also, the efficacy of a mixture of white of egg and water, in preventing or mitigating the poisonous effects of corrosive sublimate in the stomach.

Perchloride of mercury consists of

| | | | |
|---|---|---|---|
| Mercury, . . . | 1 | 200 | 73·53. |
| Chlorine, . . . . | 2 | 72 | 26·47. |
| Perchloride of mercury, | 1 | 272 | 100·00.* |

*Oxychloride of Mercury.*—"It has been stated that when a solution of corrosive sublimate, is decomposed by excess of potassa or soda, a hydrated peroxyd of mercury is thrown down, but if the mercurial salt remains in excess, the precipitate is then of a brown color, and contains oxyd and bichloride of mercury; when the alkaline carbonates are used, there are the same general results." "Chloride of mercury is not immedi-

* Turner. Davy.

ately precipitated by the bicarbonates of potassa and soda; hence that salt may be employed to detect the presence of a neutral alkaline carbonate in these bicarbonates. This oxychloride may also be formed, by passing chlorine through a mixture of water and oxyd of mercury. It may be obtained crystalline, and of a very dark color, by mixing corrosive sublimate with chloride of lime, and boiling the liquid; or, by treating a solution of corrosive sublimate with bicarbonate of potassa, and allowing the solution to stand in an open vessel, when carbonic acid gradually escapes, and the compound (hg+2c)+4(hg+2 0) is deposited. This oxychloride is decomposed by a moderate heat; chloride of mercury sublimes, and the red oxyd is left.

*Ammonia-chlorides of Mercury.*—Corrosive sublimate slowly absorbs about 4 per cent. of ammonia at common temperatures; when fused in an atmosphere of ammonia, the absorption is more rapid; the compound fuses, and consists of one atom of corrosive sublimate and one atom of ammonia. It boils at 590°, and may be distilled without decomposition, water decomposes it. (H. Rose.) When chloride of mercury, is dropped into a hot mixed solution of ammonia, and sal ammoniac, as long as the precipitate redissolves, crystals of an ammonio-chloride of mercury are formed, containing one atom of bichloride and two of ammonia. (Kane.)

When a mixture of one part of sal ammoniac and five of corrosive sublimate are dissolved in six of water, and the solution evaporated, a crystallized compound is obtained, formerly called "*sal alembroth, or salt of wisdom.*" When this solution is diluted, and mixed with solution of potassa, a white powder falls, which was considered as identical with the precipitate obtained by decomposing solution of corrosive sublimate by ammonia, but which, according to Kane, is similar in composition to the crystalline salt just mentioned, namely, (hg+2c)+2(n+3h.)"

We propose to take up next in order other compounds of mercury and simple non-metallic elements, as bromine, iodine and fluorine—which hold an *important* rank in the class of mercurial combinations; and first, that combination of mercury and bromine, known as *protobromide of mercury.* When to a saturated solution of the *protonitrate of mercury*, a saturated solution of bromide of potassium is added, precipitation immediately ensues, the result being thick and of a white color.

*Perbromide of Mercury.*—This compound crystallizes readily, under favorable circumstances, the crystals having a white color, being easily dissolved in alcohol and water, capable of fusion, and volatile. A very easy method of obtaining it is as follows—combine directly mercury and bromine; the substance in question is at once produced, or it may be obtained by using the red oxyd of mercury instead of the metal itself.

Mercury combines with iodine in such proportions as to form three distinct compounds, viz. protoiodide of mercury, *sesqui-iodide of mercury*, and *periodide of mercury.*

*Protoiodide of Mercury.*—When iodine and mercury are rubbed together in a mortar, or other suitable vessel, a small quantity of alcohol having been previously added, a powder having a very peculiar green color (dirty-green) is the result, or it may be made by precipitation, adding iodide of potassium in solution to the protonitrate of mercury perfectly neutral. It undergoes decomposition by the action of heat and light, the result being periodide, and the metal (mercury.) The following table from Brande, gives us its composition:

| | | | |
|---|---|---|---|
| Mercury, . . . | 1 | 200 | 61.5 |
| Iodine, . . . . | 1 | 126 | 38-5 |
| | — | — | — |
| Protoiodide of mercury, | 1 | 326 | 100-0 |

*Sesqui-iodide of Mercury.*—Whenever to an acid solution of the protonitrate of mercury, we add the iodide of potassium, a precipitate results which is yellowish in color (and hence has been often called the "yellow iodide of mercury.") It may be well to remark, however, that it has this color only when the process has been managed adroitly, for if the quantity of acid present be too large, the result will have a different shade of color, being a sort of mixture of the colors of the sesqui and biniodide; the latter when present, is best removed by alcohol. According to Brande, "the sesqui-iodide of mercury is more certainly obtained by precipitating the protonitrate by a sesquiiodide ef potassium, made by dissolving half an equivalent of iodine, with one equivalent of iodide of potassium; it then falls in the form of a yellow precipitate, which, when washed with alcohol, is free from periodide. This sesqui-iodide, is a yellow powder, which, by the action of dilute nitric acid, is converted into periodide, so that the precipitate which falls when iodide of potassium is added to protonitrate of mercury, varies in composition with the state of the mercurial salt; if neutral, the green protoiodide falls; if slightly acid, the yellow sesqui-iodide; if very acid, the red periodide.

This yellow compound contains, (Boullay,)

| | | | | |
|---|---|---|---|---|
| Mercury, . . . | 1 | 200 | 51-5 | 51-9 |
| Iodine, . . . . | 1½ | 189 | 48-5 | 48-1 |
| | — | — | — | — |
| Sesqui-iodide of mercury, | 1 | 389 | 100-0 | 100-0 |

*Periodide of Mercury.*—This compound may be formed by a variety of processes; when iodide of potassium in solution is added to corrosive sublimate, (also in solution,) it is abundantly precipitated, and may be collected on a filter, washed and dried; it is also obtained by rubbing together—mercury and iodine, in the following proportions, viz. mercury 1, iodine 2; again, it may be obtained, as in the first process only, using pernitrate of mercury, instead of the corrosive chloride.

Water produces little or no effect upon it, but it is soluble in alcohol and many of the acids, provided we call to our assistance a moderate amount of caloric, and when it is withdrawn, crystallization commences, and keeps pace with the progress of refrigeration. The action of ammonia upon it deserves some notice; when brought into contact with it under ordinary circumstances, ammonia is absorbed, and the red color of the compound destroyed, but ammonia being volatile, it escapes on exposure to the atmosphere, and the original red color is restored.

"When the iodide is gradually dropped into the mercurial solution, a red compound of chloride and iodide of mercury is first formed, which presently redissolves; on adding more of the iodide, a pale flesh-colored precipitate falls, which is an insoluble iodochloride; a further addition of iodide of potassium, produces a bright scarlet precipitate, which is periodide of mercury; lastly, excess of iodide of potassium, redissolves the periodide, and a soluble hydrargo-iodide of potassium is formed."*

Biniodide of mercury consists of,

| | | | |
|---|---|---|---|
| Mercury, . . . | 1 | 200 | 44-4 |
| Iodine, . . . . | 2 | 252 | 55-6 |
| Periodide of mercury, | 1 | 452 | 100-0 |

*Fluorides of Mercury.*—We mention this class only to make our series complete, for in the present state of chemical knowledge, they constitute *an unimportant class.* The substance above mentioned, is produced when peroxyd of mercury and hydrofluoric acid come into absolute contact, water dissolves the yellowish colored result. Evaporation and hot water give us as a result in this case the following: crystals which are primitive in shape and yellow in color—the compound being both insoluble and soluble, (i. e.) imperfect solution being possible, of one portion, and perfect solution of the other.

[To be concluded in the next number.]

